# Pumpkin and Pumpkin Byproducts: Phytochemical Constitutes, Food Application and Health Benefits

**DOI:** 10.1021/acsomega.3c02176

**Published:** 2023-06-23

**Authors:** Afifa Aziz, Sana Noreen, Waseem Khalid, Afaf Ejaz, Izza Faiz ul Rasool, Maham, Areesha Munir, Farwa, Miral Javed, Sezai Ercisli, Zuhal Okcu, Romina Alina Marc, Gulzar Ahmad Nayik, Seema Ramniwas, Jalal Uddin

**Affiliations:** 1 Department of Food Science, Faculty of Life Sciences, Government College University, Faisalabad 38000, Pakistan; 2 University Institute of Diet and Nutritional Sciences, Faculty of Allied Health Sciences, The University of Lahore, Lahore 54000, Pakistan; 3 University Institute of Food Science and Technology, The University of LahoreLahore 54000, Pakistan; 4 College of Biosystem Engineering and Food Science, Zhejiang University, Hangzhou 310027, P.R. China; 5 Department of Horticulture, Faculty of Agriculture, Ataturk University, 25240 Erzurum, Türkiye; 6 HGF Agro, Ata Teknokent, 25240 Erzurum, Türkiye; 7 Department of Gastronomy, Faculty of Tourism, Ataturk University, 25240 Erzurum, Türkiye; 8 Food Engineering Department, Faculty of Food Science and Technology, University of Agricultural Sciences and Veterinary Medicine, 400372 Cluj-Napoca, Romania; 9 Technological Transfer Center “CTT-BioTech”, University of Agricultural Sciences and Veterinary Medicine Cluj-Napoca, Calea Floreşti Street, No. 64, 400509 Cluj-Napoca, Romania; 10 Department of Food Science & Technology, Govt. Degree College, Shopian-192303, J&K, India; 11 University Centre for Research and Development, Chandigarh University, Gharuan, Mohali 140413, Punjab, India; 12 Department of Pharmaceutical Chemistry, College of Pharmacy, King Khalid University, Asir 61421, Saudi Arabia

## Abstract

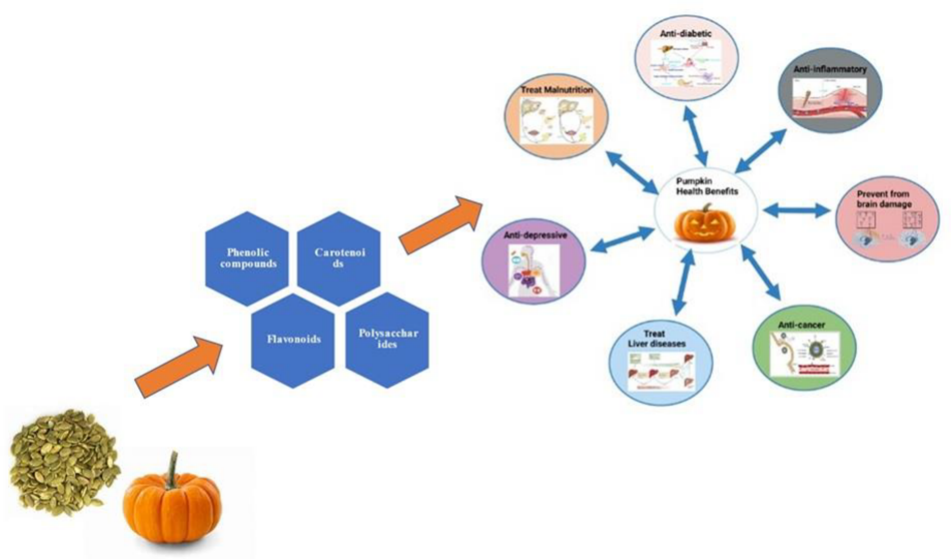

Nowadays, agricultural waste byproducts are exploited in the food industry rather than discarded. Pumpkin is one of the most significant vegetable crops that is widely consumed in farmland and certain urban regions. The current study was designed to measure the phytochemical constituents, food application, health benefits, and toxicity of pumpkin and pumpkin byproducts. Pumpkins and pumpkin byproducts (seeds, leaf, and skin/peel) can be utilized as functional ingredients. Different parts of the pumpkin contain bioactive compounds including carotenoids, lutein, zeaxanthin, vitamin E, ascorbic acid, phytosterols, selenium, and linoleic acid. Pumpkin is used in various food sectors as a functional food, including baking, beverages, meat, and dairy industries. Furthermore, the leaves and pulp of the pumpkin are used to produce soups, purees, jams, and pies. Different parts of pumpkins have several health benefits such as antidiabetic, antioxidant, anticancer, and anti-inflammatory effects. Therefore, this review paper elaborates on the pumpkins and pumpkin byproducts that can be used to develop food products and may be valuable against various diseases.

## 1 Introduction

One of the biggest problems faced by the food industries is waste generation, which results in a great amount of food byproducts.^1^ It is considered a major global challenge from all points of view and represents the inefficient use of natural resources. One third of the produced food in the world is wasted. For this reason, the evaluation of food byproduct applications has been the focus of many researchers to minimize these problems.^2^

Pumpkin (*Cucurbita moschata Duch ex Poir*) is one of the most significant vegetable crops of Mexico and is widely cultivated in South Asia, Africa, India, Latin America, and the United States. Although pumpkins have long been consumed in farmland and certain urban regions, horticultural, commercial, industrial, and scientific studies now examine them in more detail.^3^ Pumpkin varieties include *Telfairioccidentalis*, *Moschata Cucurbita*, *Pepo Cucurbita*, *Maxima Cucurbita*, and *Cucurbitamixta*. The most popular pumpkin varieties worldwide are *Cucurbita pepo*, *Cucurbita maxima*, and *Cucurbita moschata*.^4^ Pumpkin also produces a lot of byproducts such as pumpkin seeds, shells, peels, and skin, which are mostly discarded by households. Effective utilization of pumpkin byproducts involves extracting bioactive components and adding them to the food industry for enhanced nutritional value. Pumpkin contains various bioactive compounds, such as carotenes, lutein, zeaxanthin, vitamin E, ascorbic acid, phytosterols, selenium, and linoleic acid, which operate as antioxidants in the human diet. The different parts of pumpkins are great sources of functional ingredients.^5^ Pumpkin is employed in various sectors as a functional food, including baking, drinks, meat, and dairy. Pumpkin flour may also increase the gluten network in the dough, which helps the bread rise and stabilizes the gas cell structure. However, these factors also help to improve bread nutritional and functional qualities.^6,7^

The pumpkin and different parts of the pumpkin can be used in industrial applications as a functional ingredient. The use of fresh or dried components obtained from the pumpkin in meat products such as pumpkin meals^8^ has been the subject of several studies.^9^ However, there has not been any research on using flour and pumpkin seed mixes in beef products. The major items made from pumpkin fruit are juices, pickles, and dried goods. Pumpkin juice is a drink that serves a purpose and is prepared from byproducts like sweetened whey, sweetened buttermilk, and whey that contains pumpkin pulp.^10^ Worldwide, the leaves are also consumed as vegetables, and the pulp is used to produce soups, purees, jams, and pies. Pumpkin is thought to provide several health advantages due to its range of bioactive components including antidiabetic, antioxidant, anticancer, and anti-inflammatory effects.^11^ It has reportedly been used therapeutically to cure tapeworms, schistosomiasis, and ascariasis. In this background, the purpose of this review article was to review the published advances concerning the incorporation of pumpkin and pumpkin byproducts in the food industry. Moreover, this study elaborates on the nutritional profile, pharmacological aspects, health perspectives, and industrial applications of pumpkins.

## 2 Phytochemicals in Pumpkin

Phytochemicals are organic substances derived from plants that have physiological effects that benefit humans in terms of nutrition and medicine.^12^ However, they also enhance plant color, scent, and flavor by guarding them against illness and other negative consequences. Plant components that shield plants against environmental dangers including pollution, stress, dehydration, UV radiation, and disease attack, are collectively called phytochemicals.^13^ The current study showed that high consumption of pumpkin can preserve human health from different diseases.^14^ Phytochemicals are constitutive metabolites that control critical development and reproductive processes while allowing plants to tolerate short-term or long-term environmental stresses.^15^ Phytochemicals are often categorized as main or secondary metabolites based on their role in plant metabolism. The common metabolites include common sugars, amino acids, proteins, and nucleic acids as well as chlorophyll, purines, and pyrimidines present in pumpkins. Secondary metabolites relate to the residual plant substances, which include curcumin, saponins, phenols, glucosides, terpenes, flavonoids, lignans, plant steroids, and flavonoids.^6,7^ Pumpkin seed oil is a significant source of phenolic compounds which have drawn significant scientific interest due to their potential health benefits (because they contain hydroxyl functional groups capable of scavenging free radicals and are well-suited to reduce the risk of several oxidative degenerative diseases).^13,16^

Pumpkin is a rich source of health-promoting antioxidants, polyphenols, and carotenoids.^17,18^ According to studies, a diet rich in antioxidants reduces the incidence of diabetes, cancer, and cardiovascular disease.^19^ Antioxidant chemicals (pumpkin seeds) can help lower blood sugar levels in animals with impaired glucose metabolism.^17^ Antioxidant intake is associated with a decreased risk of neurodegenerative diseases like Alzheimer’s.^20^ Additionally, oxidative stress is brought on by insufficient antioxidant levels in the body, which is linked to the emergence of depression.^13^ Therefore, it is crucial to incorporate meals with pumpkin pulp.^21^ Carotenoids, lutein, zeaxanthin, vitamin E, ascorbic acid, phytosterols, selenium, and linoleic acid are among the beneficial compounds in pumpkin that serve as antioxidants in the human diet. Delicious ripe squash contains carotene-rich orange or yellow flesh.^22^ Since pumpkin flesh is rich in fiber, vitamin C, vitamin E, magnesium, potassium, and other carotenoids, it is a terrific source of these amazing phytonutrients. The body converts one of the plant chemicals known as carotenoids into vitamin A. Carotenoids play several functions in general health by assisting in the metabolism of vitamin A, which has been shown to reduce the incidence of colon and lung cancer. It is most beneficial when combined with other carotenoids.

**Figure 1:**
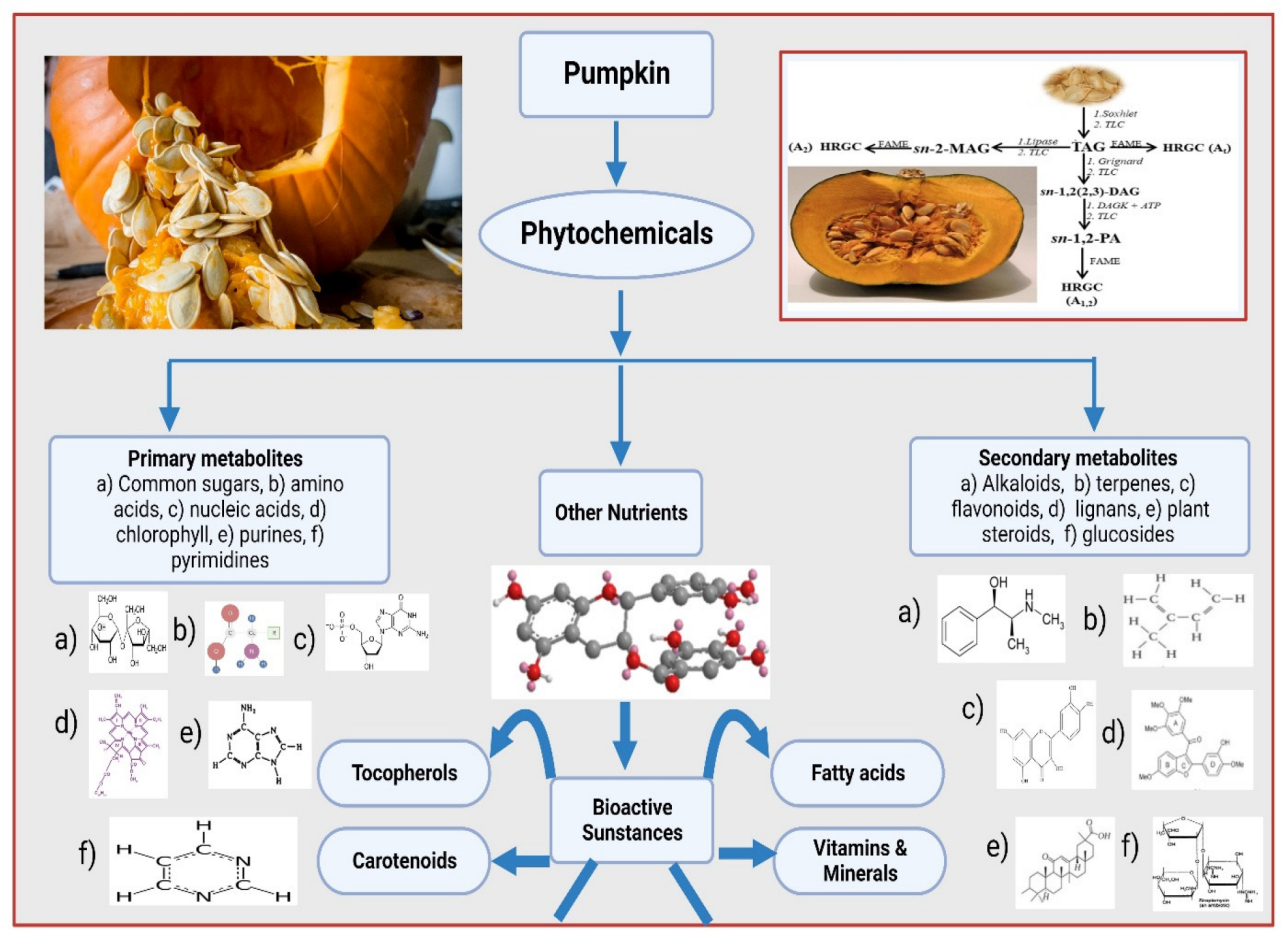
Photochemistry of pumpkin.

Pumpkin is a powerful antiaging tool that fights melanoma, cataracts, and other diseases. It also has a large amount of carotene in it. Pumpkin is vitamin-rich, low in fat and salt, and devoid of cholesterol (Figure 1). Carotenoids are crucial to avoid dry eye disease. Both pumpkin and pumpkin seeds contain several necessary components. Seeds are low in sodium and abundant in calcium (Ca), manganese (Mn), phosphorus (P), and magnesium (Mg). They are also a great source of trace elements including copper, iron, zinc, manganese, and iron. Some minerals have antioxidant properties that function as cofactors for antioxidant-dependent biocatalysts.^23^ Similarly, pumpkin seeds with high potassium and low salt content have substantial therapeutic benefits in enhancing cardiovascular health, male reproduction, structural proteins, and cell defense. The mineral content of pumpkin seeds makes them a good diet.^24^ Different types of pumpkin phytochemicals are listed in Table 1.

**Table 1:** Phytochemicals in Different Parts of Pumpkin

Pumpkin part	Extraction method	Phytochemicals	References
Seed of pumpkin	Chromatography	Fatty acids like palmitic, stearic, oleic and linoleic acids, sulfur-containing amino acids, and phytosterols	Ahmad and Khan^3^
Pumpkin	–	Carotenoids, alkaloids, flavonoids, polyphenols, tannins, tocopherols, phytosterols, and cucurbitacin	Kaur^25^
Pumpkin pulp	HPLC analysis	Carotenoids, phenolic acids, flavonols, minerals, and vitamins	Kulczyński and Gramza-Michałowska^6^
Pumpkin fruit	HPLC analysis	Carotenoids, polyphenols (flavonols and phenolic acids), tocopherols, minerals (K, Ca, Mg, Na, Fe, Zn, Cu, Mn), and vitamins (C, B1, folates)	Silva^26^
Fresh pumpkin	Spectrophotometer	Acorbic acid content, polyphenols and carotenoid content	Datta^27^
Pumpkin oil	DPPH, ABTS and reducing power	Chlorophyll b and total carotenoids	Can-Cauich^28^
Seed of pumpkin	Spectrometer	Carotenoids	Veronezi^29^
Pumpkins	–	Carotenoids, terpenoid-metabolites	Montesano^30^
Pumpkin seed oil	Spray-drier	Tocopherols, squalene, and sterols	Ogrodowska^31^
Pumpkin peel, flesh and seeds	–	Pectin, polysaccharides and fiber, essential oils, proteins, phenols and minerals, carotenoids	Hussain^32^
Pumpkin (seeds and shell)	HPLC and U.V. Spectrophotometer	Phenolic content and antioxidants	Saavedra^18^
Seeds of pumpkin	Spectrophotometer	Carotenoids	Wongsagonsup^33^
Pumpkin waste	Freeze-drying encapsulation	β-Carotene, phenolics	Rezig^34^
Pumpkin seed oils	Cold pressing and solvent extraction methods	Tocopherols, sterols, β-carotene, and lutein	Stajčić^35^
Pumpkin	Ultrasound-assisted extraction (UAE)	Phenolic compounds	Atallah^36^
Pumpkin flower	UV/Visible Spectrophotometer	Phenol, flavonoid, antioxidant, and anthocyanin	Ghosh^37^

## 3 Food Application of Pumpkin

Food is a basic necessity of all living organisms. In normal life, we consume food in raw and processed forms. Nowadays, we mostly consume artificial food that has some side effects. Industrialist and food technologists are currently trying to develop functional foods. The industrial food applications of pumpkins are shown in Table 2.

**Table 2:** Industrial Applications of Pumpkin

Industry	Food product	Additive	Function	Reference
Meat	Beef meat balls	Pumpkin seed kernel flour	Fat replacement	Longato^38^
	Chicken burgers	Pumpkin seed	Enhanced stability during storage	Öztürk^39^
	Low-fat meat balls	Pumpkin flour, wheat germ, and date seed powder	Fat replacement	Ammar^8^
	Rice sponge cake	Pumpkin flour	Loaf volume decreased with the increase in pumpkin flour	Kessler^40^
	Meat	Pumpkin and pork fat	Reduced fat, lightness of meat batter, and increased chewiness	Kim (41)
	Beef patties	Pumpkin seed and pulp	No change in textural property and decrease moisture content	Serdaroğlu^42^
	Horse meat patties	Pumpkin	Increase vitamin C and A content	Abilmazhinova^43^
	Meat cutlets	Pumpkin carrot powder	Increase the content of carbohydrates and improve organoleptic characteristics (juiciness, consistency, smell, and taste)	Kassymov^44^
	Beef	Pumpkin powder	Increase emulsion stability	Unal^45^
	Fish	Pumpkin	Enhance color quality of koi fish	Ayi^46^
Bakery	Bread	Pumpkin flour	Good nutritional value as it is high in ash, fiber, and β-carotene contents	Wongsagonsup^33^
	Cake	Pumpkin oil	Facilitate gastrointestinal digestion	Čakarević^47^
	Biscuits	Pumpkin seed flour and refined wheat flour	Decrease serum blood glucose level	Malkanthi^48^
	Biscuits and cookies	Pumpkin seed flour and wheat flour	Prevent constipation, diabetes, prolong intestinal transit time, and lower cholesterol level	Kumari^49^
	Cookies	Pumpkin powder and wheat flour	Low fat and reduced carbohydrates	Anitha^50^
	Muffins	Pumpkin powder	Increase nutritive value	AlJahani and Cheikhousman^51^
Beverage	Juice	Pumpkin	Increased health-promoting characteristics and improved the sensory quality of the products	Mala^52^
	Pineapple juice	Pumpkin pulp	Reduce constipation	Adubofuor^53^
	Smoothies	Pumpkin paste	Give nutrition to human body	Eid^54^
	Mango juice	Pumpkin paste	Promote health	Kidoń^55^
Dairy	Strawberry	Pumpkin pulp	Enhance immune system	Atallah^36^
	Cereal milk	Pumpkin pulp	Act as a functional food	Shendge and Patharkar^56^
	Yogurt	Pumpkin pulp	Increase health benefits	Barakat^57^
	Ice cream	Pumpkin product	Retain natural color and photochemical health benefits	Hassan and Barakat^58^
	Ice cream	Pumpkin seed	An increase in the protein content improves the degree of satiety and enhances sensory characteristics	Soleimanian^59^
	Curd mass	Powder from pumpkin pulp	Increase quality characters and expand the assortment line of curd products with a functional load	Babukhadia^60^

### 3.1 Application in the Meat Industry

The flesh of an animal that is eaten as food is known as meat. The word “meat” originates from the Old English word “mete,” a term for an ordinary meal. The phrase also has roots in the Danish, Swedish, and Norwegian words mat and mad, which similarly mean “to eat.” People of all ages from different cultures like various ground beef meals, including meatballs and burgers.^61^ However, these meat products also have certain undesirable characteristics.^62^ Eliminating fat from meat products has several unfavorable repercussions on sensory and technical levels, including reduced yields, higher cooking losses, and unstable emulsions. Additionally, the taste and juiciness are lost due to changes in texture.^63^ A study examined the effects of adding pumpkin seeds to chicken patties and suggested that these ingredients could improve both the baking and lipid oxidation aspects of the final product. A previous study discovered that replacing red meat in beef patties with a combination of dry pumpkin seeds and flour was an acceptable substitution that harmed the patty texture and increased water retention.^39^ It substitutes powder for fat to make beef balls.^39^ It was claimed that the cooking properties survived, although without the excellent characteristics of the hamburger. Additionally, using fat vegetable alternatives such as soybean oil and pumpkin seed meal is a practical option.^64^ Beef pellets were prepared using pumpkin seed meal rather than beef fat to provide a more nutrient-dense and practical product. The fatty acid content and health index evaluate its nutritional worth, while sensory assessment is used to measure consumer happiness.

### 3.2 Application in the Bakery Industry

Pumpkin is often eaten raw, boiled, steamed, or mixed with soups and curries. Pumpkin is rich in β-carotene, which has an orange or yellow color and acts as an antioxidant. It is used in bakery products, such as sandwiches, delicious cookies, buttercream, and muffins. Instead of flour, pumpkin flour can in used in bread products in different ways.^65^ Pumpkin flour is the main ingredient that is used in baking products. However, it contains small amounts of β-carotene, vitamin A, and other phytochemicals.^66^ The pumpkin replacement sample contents were observed along with their physical, chemical, and sensory properties. The physical texture and feel of the finished goods were negatively impacted by the substitution of pumpkin pulp in the sandwich, cake, and cookie recipes by more than 15%, as opposed to 20% in the butter and chiffon cake recipes. The alternative uses of pumpkin including pumpkin paste, bread mixes, and baked goods have many more calories and are fortified with vitamin A, and pumpkin powder is the greatest source of nutrients and antioxidants. Overall, people surveyed showed that the pumpkin-based products were acceptable, and they would buy the items.

### 3.3 Application in the Beverage Industry

Functional juices of fruits and vegetables have been produced to meet the demand of the beverage industry. The blending of two or more different kinds of fruits/vegetables produces a healthy juice which can overcome the single-component juice demand. The blended juice represents high-quality consistency and enhanced nutritional or phytochemical properties,^53^ and interest has been developed to research and produce blended juices. A study was performed to develop a pumpkin pulp and pineapple blended juice. The finding of the study showed the improved physicochemical and sensory properties of juice blends.^53^ Another study was conducted to produce pumpkin pulp-fortified juice enriched in vitamins, antioxidants, and minerals. The outcomes of the study proved that mixing pumpkin juice with mango and strawberry juice enhanced the sensory quality.^51^ Interestingly, the blended pumpkin juice produced in the study gained better acceptance by children and the elderly, and the research revealed that pumpkin has the ability to produce functional juice.^51^

### 3.4 Application in the Dairy Industry

Scientists are investigating the alteration of the physicochemical properties of milk, yogurt, and ice cream. A study was performed to observe the prospect of producing a new kind of ice cream product with the addition of a substantial amount of pumpkin pulp. The results showed that the ice cream produced had a moderate amount of fat and better textural and emulsification properties.^57^ In addition, another study was performed to investigate the addition pumpkin seed powder in cereal milk fermented with *Lactobacilli* and *Bifidobacteria* cultures for the development of nondairy probiotic products.^56^ The results revealed that the fortification of pumpkin seed powder improved the sensory and physicochemical properties of cereal milk. Moreover, the shelf life of cereal milk was enhanced to 9 days under refrigerated storage.^56^ Hence, the findings of the studies showed the effective utilization of pumpkins byproducts in the dairy industry.

## 4 Sensory Acceptance of Pumpkin Addition Products

A versatile vegetable like pumpkin may be found in various dishes and drinks, including dairy products, cakes, muffins, and morning cereals. Pumpkin byproducts are used more frequently in food processing because of their high nutritional value and possible health benefits.^25^ Increased efforts for the sustainability of the environment drives demand for wholesome and reasonably priced food, which contributes economic value to manufacturing and aids in the creation of goods. Pumpkin waste is managed by the formation of innovative products.^67^ The results revealed the number of vitamins, minerals, and phytonutrients in pumpkin waste byproducts; for example, pumpkin pulp is used in bakery items as a source of carotene, vitamin A, and fiber.^51^ A low-fiber diet may cause constipation and other gut issues among children. For improving gut health, a high-fiber diet plays an important role. Pumpkin pulp weaning is also a great fiber source which is very beneficial for baby gut health.^63^

**Figure 2:**
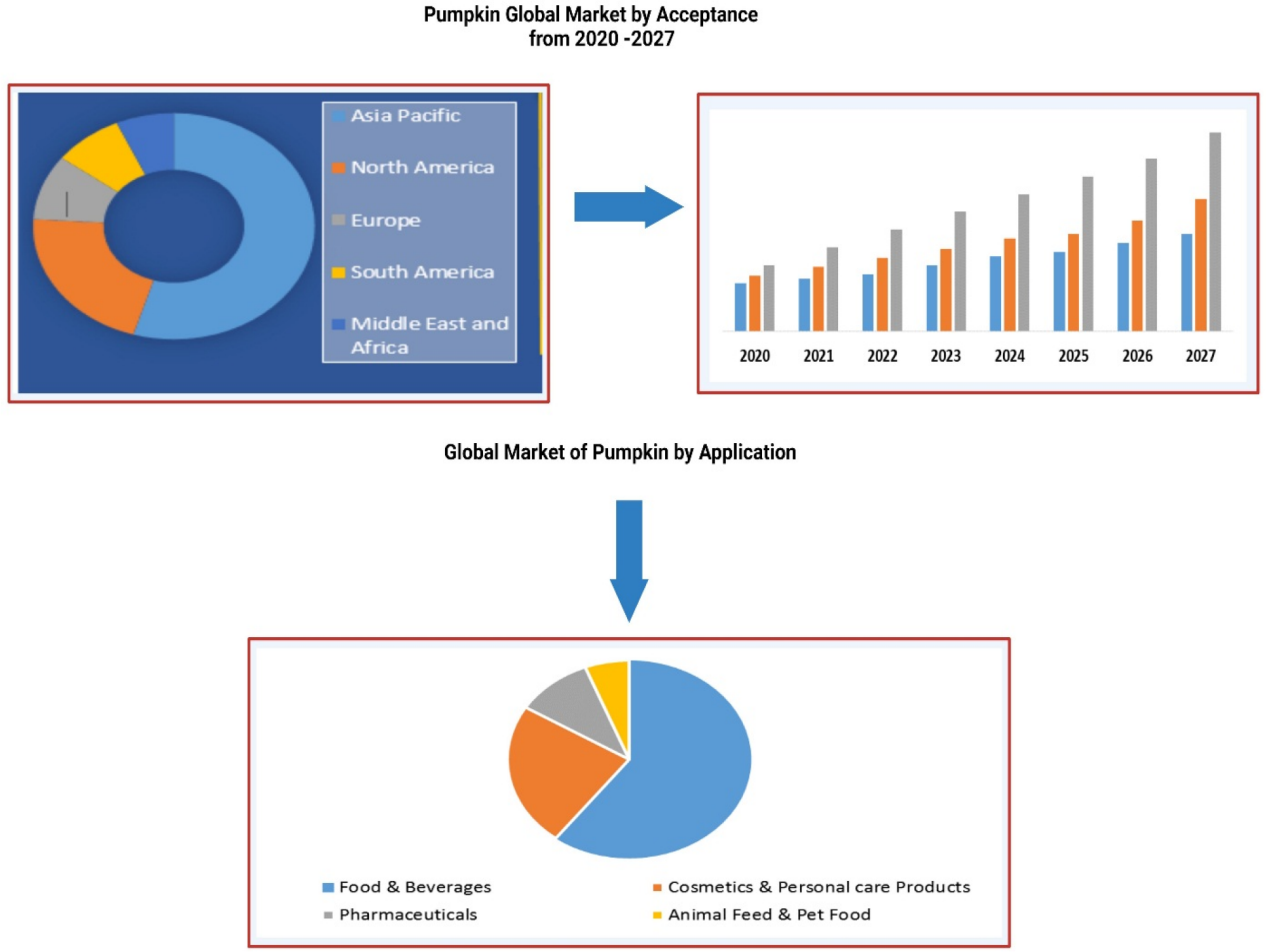
Consumer acceptance of pumpkin.

Pumpkin skins are rich in protein, vitamins, minerals, and fiber.^68^ Although pumpkin juice is incredibly healthy, teens and older people often do not like its flavor. Pumpkin juice was blended with other fruit liquids to enhance the flavor and fragrance. The aesthetic appeal of the beverage was greatly enhanced when pumpkin juice was combined with orange and strawberry juice. Surprisingly, the combination of fruit and pumpkin juice has been well-received by youngsters and older people, which may encourage people to drink it as a healthy juice (Figure 2).

## 5 Health Benefits of Pumpkin

The pumpkin has various uses for both humans and animals because it is a potentially beneficial food source. It has been demonstrated that phytochemicals directly affect nutrient-dense foods. For example, dietary fiber can manipulate the glycemic response that reduces the risk of diabetes.^69^ One of the common ailments among elderly individuals is diabetes. Diabetes mellitus is a metabolic illness in which the body either does not make insulin or does not have enough. The are two kinds of diabetes, including type I diabetes and type II diabetes. Pumpkin seeds and pumpkins contain substances that assist in decreasing blood sugar, and many diabetics believe eating pumpkins will not hurt them because pumpkins have a high fiber content.^70^ Various bioactive substances are also present, including polysaccharides, *para*-aminobenzoic acid, fatty acids, sterols, proteins, and peptides. Furthermore, it is also a reliable supply of γ-aminobutyric acid. Pumpkin seeds (*Cucurbita* spp.) are regarded for their substantial linoleic acid, important fatty acid, and high protein content. The pumpkin seeds contain amazing amounts of vital amino acids. Additionally, pumpkin seeds have many important trace elements, including K, Cr, Na, Mg, Zn, Cu, Mo, and Se.^71^

The abnormal growth of cells is called cancer. A major challenge for researchers and professionals is selecting prevention and treatment strategies to prevent and cure cancer. A variety of fruits and vegetables, including pumpkin seeds, can help to reduce cancer risks. Pumpkin seed oil contains high levels of various carotenoid pigments, which have been shown to reduce the risk of cancer. It has been reported that the risk of various cancers (breast, rectal, and lung cancer) is inversely associated with pumpkin seed consumption.^72^

Malnutrition is a global problem affecting children whose calorie and protein intake is restricted. Malnutrition often leads to behavioral problems. Protein–energy malnutrition (PEM) has been reported to lead to the generation of free radicals through lipid peroxidation.^73^ Lipid peroxidation is a risk factor associated with brain injury. The generation of free radicals, such as reactive oxygen species (ROS), damages brain cells and leads to serious side effects of PEM. In a recent study, pumpkin leaves were used to investigate the brain-protective effects of herbs in PEM-induced rats due to their high antioxidant content. The seed protein and the ribbed squash leaves were assembled to prevent PEM-induced oxidative damage to brain cells.^74^

Organic compounds known as aflatoxins have significant toxic effects including carcinogenicity, mutagenicity, and hepatotoxicity. They also contribute to lipid peroxidation and affect the brain.^75^ It has been reported that pumpkin seed oil treats adverse effects on brain tissue caused by aflatoxin.^76^ Depression is the most common brain disorder described as a disruption of interests, desires, sleep, and eating habits. Mood swings can also make a person feel guilty or ashamed. Affected individuals are also less interested and focused on daily work.^40^ Pumpkin leaves were reported to be useful in treating depression and seizures, especially due to their muscle-relaxant properties in hydroethanolic leaf extract.^77^ Another study suggested that the antidepressant effects of pumpkin can help treat depression.^78^ Pumpkin seeds contain high levels of tryptophan (576 mg per 100 g) in the form of serotonin (a neurotransmitter), which helps fight depression. A study was performed to evaluate the antidepressant properties of pumpkin seeds. The effects of raw and processed pumpkins were evaluated by inducing depression in rats by injection of methyl isobutyl ketone. The effects of natural and processed squashes were evaluated. These two pumpkin seed extracts are believed to have antidepressant effects and are an alternative to antidepressants, which have side effects.^79^

**Figure 3:**
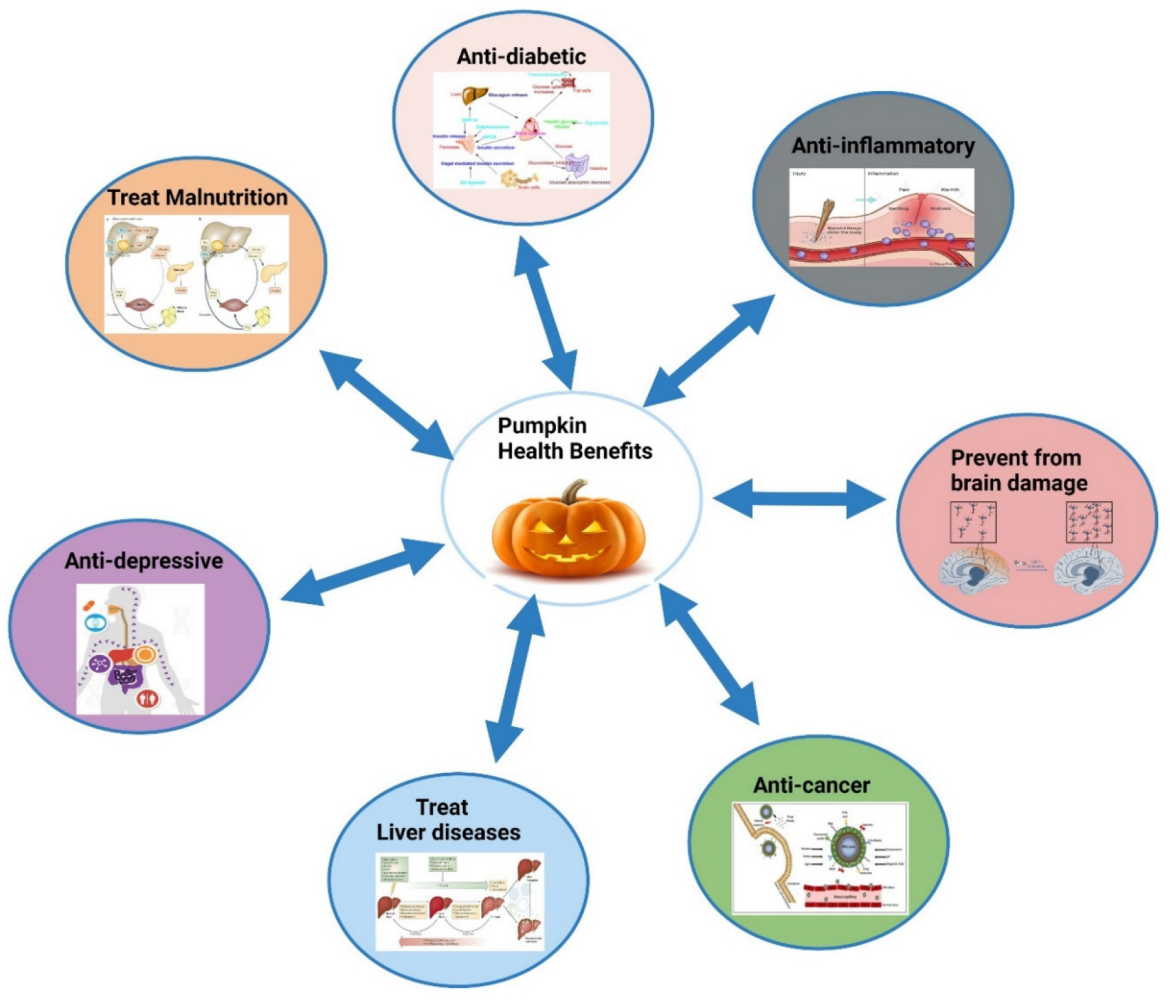
Therapeutic potential of pumpkin.

The liver is an important organ in our body. It performs many important functions, such as synthesizing proteins, detoxifying various metabolites, and producing essential biochemicals important for the digestive process. It also plays an important role in regulating glycogen storage, metabolism, hormone production, and red blood cell breakdown. The liver also plays an important role in fat metabolism. It is responsible for adipogenesis, cholesterol synthesis, triglyceride production, and lipoprotein synthesis.^80^ Nonalcoholic fatty liver disease (NAFLD) is a chronic disease that presents a broad spectrum of pathology, including simple fatty liver infiltration.^81^ The effects of pumpkin seed-rich biscuits (*Cucurbita*) containing 15% flour and 3% oil on the liver induced by amitriptyline in laboratory rats were measured. A previous study reported that treatment with biscuits made from 15% pumpkin seed meal and 3% pumpkin seed oil biscuits reduced serum cholesterol (TC), triglycerides (TG), high-density lipoprotein (HDL-c), and lipoprotein low density (LDL-c), while serum very low-density lipoproteins (VLDL-c) were associated with decreased serum AST and ALT activity. The results showed that 15% pumpkin seed meal cookies and 3% pumpkin seed oil crackers exhibit different properties, including total antioxidant, superoxide dismutase (SOD), weight gain, feed intake, feed efficiency, and an increase in HDL-c. In addition, nitric oxide (NO) levels were reduced compared with positive control mice^44^ (Figure 3 and Table 3).

**Table 3:** Therapeutic Properties of Different Pumpkin Parts

Pumpkin part	Disease	Recovery	References
Pumpkin flour	Hypertension and oxidative stress	Control of glucose absorption and reduction of associated hypertension	Vergara-Valencia (82)
Pumpkin seed oil	Cardiovascular problems of menopausal women	Functional food used as culinary and traditional medicine	Šamec^24^
Pumpkin fiber	Gastrointestinal parasites, urinary dysfunctions and benign prostatic hyperplasia (BPH) as a supporter, dysuria, CVDs and maintenance of blood glucose	The fibers in the pumpkin are useful in the buffering of stomach pH by binding the excess acids produced by the digestive system	Vergara-Valencia^83^
Pumpkin seed oil	Diastolic blood pressure in postmenopausal women	Reduced the risks of heart attacks because of high magnesium content	Matsuzaki^84^
Pumpkin seed	Hypertension	Relaxing vessels on chemical-induced hypertension	El-Mosallamy^85^
Pumpkin seed	Diabetes	Increased plasma enzyme levels	Matsuzaki^86^
Pumpkin seed oil	Liver disease	Depletion of cholesterol synthesis and elevated cholesterol catabolism in the liver	Al-Okbi^87^
Pumpkin	Tumor and cancer	Removal of various free radicals generated in the body during metabolism	Chen and Huang^88^
Pumpkin pulp and oil	Lung histomorphological damage	Antimicrobial activity	El-Aziz^89^
Pumpkin	Diabetes, carcinogenicity, and inflammation	Various medical conditions for the cure	Yadav^67^
Pumpkin seed powder	Diabetes	Pumpkin seed supplementation significantly normalized the alterations of different biochemical parameters of diabetic mice	Arzoo^90^
Pumpkin	Diabetes and oxidative stress	Therapeutic strategies have recently focused on preventing such diabetes-related abnormalities using different natural and chemical compounds	Shayesteh;^91^ Makni et al. (92)

## 6 Clinical Trials (Human and Animal Studies)

According to Kim et al.,^93^ clinical trials have established a correlation between the consumption of pumpkin and β-carotene among patients with depression and an increase in the levels of norepinephrine and serotonin in the brain, which are in control for alleviating depression. Choosing the right diet rich in essential micronutrients can strengthen the body’s adaptive immunity, preventing attacks from pathogens. The significance of maintaining a healthy diet in the fight against infectious diseases cannot be overstated. Pumpkin, its flesh, peel, and seed powders are abundant in crude fiber and proteins, particularly pumpkin seed proteins containing peptides vital in promoting healthy human body functions.^94^ A study conducted by Quanhong et al.^95^ revealed that the hypoglycaemic effects of pumpkin are attributed to polysaccharides extracted from the pumpkin fruits. The study evaluated the hypoglycemic activity of these polysaccharides on alloxan-mediated diabetic rats, where improved levels of blood insulin and decreased blood glucose levels were observed, indicating better glucose tolerance. Additionally, another study by Fahim et al.^96^ testified on the anti-inflammatory activity of pumpkin, with pumpkin seed oil inhibiting adjuvant-induced arthritis in rats for effective arthritis treatment. When used as a formulation with standard drugs, natural substances from pumpkin can enhance the anti-inflammatory action. Furthermore, researchers also demonstrated that pumpkin fruit extracts significantly increased the actions of glutathione peroxidase and superoxide dismutase, while reducing the concentration of malonaldehyde in mice. Moreover, glutathione peroxidase and superoxide dismutase were more noticed by pumpkin polysaccharides in the serum of tumor-containing mice.^97^

## 7 Drug–Pumpkin Bioactive Interaction

Traditional and indigenous drugs hold unusual meaning as they have been tested over a long time and are comparatively safe, easily available, and inexpensive.^98^ Many of these have been used as dietary adjuncts to treat chronic and severe diseases. Combining natural components of pumpkin and its byproducts with standard drugs may result in synergistic, antagonistic, or insignificant effects, known as drug interaction effects, for treating diseased conditions. Pumpkin and its byproducts have also proven useful in treating several diseases alongside drugs. Diabetes is becoming more prevalent, resulting in a significant economic burden. The scientific community is under pressure to develop safer and more cost-effective treatments for this disease. Herbal medicines have been identified as potential treatments. As a result, recent studies have focused on the antidiabetic properties of herbal formulations, including pumpkin.^99^ Pumpkin is a commonly cultivated plant, and its fruits can be used as dietary supplements for individuals with diabetes. For instance, a formula consisting of pumpkin along with chicken and rice has been proven to provide benefits to children with diarrhea.^100^ Al Zuhair et al.^101^ proposed that pumpkin seeds contained hypotensive activity. When verified on animal models with standard hypotensive drugs such as felodipine, pumpkin seed oil exhibited a good drug interaction effect.

## 8 Molecular Docking

Pumpkin seed has a protein content of up to 65%.^102^ Recent studies have recognized pumpkin seed protein as a source with functional properties, including good digestibility, solubility, emulsifying properties, and foaming properties.^102^ Studies have reported on the covalent interactions between protein hydrolysates and pyrogallic acid, which depend on the free amino groups of the protein.^103^ Studies have demonstrated the various alterations in the molecular structure and mechanisms of PSP as it interacts with flavonoids such as apigenin. The binding process between PSP and apigenin was primarily facilitated by hydrophobic interactions, which fostered modifications in the conformation, microenvironment, and surface hydrophobicity of the protein. Molecular docking analyses and molecular dynamic (MD) simulations illustrated the consistent binding of apigeninobic pockets.^104^ These findings suggested that the molecular interaction of pumpkin protein and polysaccharides with other bioactive components can be used to achieve functional proteins.

## 9 Structural–Activity Relationship

The functional and biological activities of pumpkin active compounds are strictly related to their structural characteristics; therefore, it is important to understand the correlation between their biological activities and structures. One study isolated a polysaccharide from pumpkin powder and investigated its structural features by using partial acid hydrolysis using NMR and Fourier transform-infrared spectroscopy (FTIR). The results showed that the polysaccharide primarily consisted of (1–6)-α-Galp, (1–4)-α-Glcp, and (1–4)-β-Galp in different ratios.^105^ In another study, a polysaccharide was extracted from the pumpkin seeds. The backbone contained (1–4)-linked β-d-glucopyranosyl, (1–6)-linked α-d-mannopyranosyl, and (1–2)-linked α-d-galactose.^105^ PSP-I also demonstrated restrained scavenging activities against DPPH and OH radicals, with a dose-dependent effect against DPPH radicals.^106^ Another study was performed, which showed that pumpkin polysaccharides at a concentration of 200 mg/mL showed antibacterial activities against *Staphylococcus aureus*, *Listeria monocytogenes*, and *Escherichia coli*.^107^

## 10 Toxicity

A study investigated the potential acute and subacute toxicity of the hydroalcoholic extract from *C. maxima* seeds in mice to examine any possible toxic effects on specific organs. The findings indicated that the extract was safe and free of acute toxicity at a dose of 5000 mg/kg.^108^ On the other hand, certain essential oils have been shown to have multiple pharmacological properties and therefore represent a promising area of focus for pharmaceutical sciences. In one particular study, the ability of pumpkin seed oil (PSO) to improve methotrexate-mediated lung toxicity in rats was investigated. Lung tissue analysis showed that PSO could decrease malondialdehyde levels, enhance glutathione and nitric oxide levels, increase cholinesterase activity, and reduce tumor necrosis factor-α.^109^ Another study examined the defensive effect of pumpkin seed extract against escitalopram-mediated reproductive toxicity in male mice. Reproductive toxicity was observed in mice treated with 10 or 20 mg/kg escitalopram for 30 and 60 days. However, coadministration of escitalopram oxalate (10 or 20 mg/kg) with pumpkin seed extract (300 mg/kg) was found to attenuate the testicular toxicity induced by escitalopram.^110^

## 11 Conclusion and Future Prospects

It is concluded that pumpkin is an important vegetable crop widely consumed in different regions of the world. Pumpkin has gained attention due to its nutritional profile, pharmacological aspects, and industrial use, which may be due to the immense number of phytochemicals and bioactive compounds. Pumpkin byproducts are also used in several food products as a functional ingredient. Different parts of the pumpkin are composed of bioactive compounds that can reduce the risk of several chronic diseases. Thus, it is mandatory to explore the importance of pumpkins for consumers due to their phytochemical profile, health aspects, and industrial applications. Further studies are needed to identify the additional bioactive profile and potential industrial applications of pumpkins.

The bioactive compounds of pumpkin and pumpkin byproducts, which are high in phytochemicals, can prevent the oxidation process in different foods. It should also be remembered that functional foods can provide their possibly subtle benefits. To prove that pumpkin byproducts have functional effects on different food products, recent processing techniques should be used. Future randomized control trials (RCTs) should seek to compare the effects of a control diet and a pumpkin-based intervention diet on the biomarkers of chronic disease. The impact of the whole diet, reflecting synergy between components, needs to be measured, because most previous studies have focused on different extracts and components. Regardless, studies can be performed on how pumpkin byproducts can be consumed in households. Nonetheless, results obtained from different types of experimental studies contribute to a more complete understanding of how the pumpkin byproduct nutritional matrix may be beneficial.
