# Correction to “Pumpkin and Pumpkin Byproducts: Phytochemical Constitutes, Food Application and Health Benefits”

**DOI:** 10.1021/acsomega.5c00201

**Published:** 2025-09-01

**Authors:** Afifa Aziz, Sana Noreen, Waseem Khalid, Afaf Ejaz, Izza Faiz ul Rasool, Maham, Areesha Munir, Farwa, Miral Javed, Sezai Ercisli, Zuhal Okcu, Romina Alina Marc, Gulzar Ahmad Nayik, Seema Ramniwas, Jalal Uddin

The figure legends for Figures 1 and 3 are being corrected to add appropriate credit information for reused images:

Figure 1: Photochemistry of pumpkin^1^ (adapted from ref 1 published by MDPI 2018 under an open access Creative Common CC BY license).

Figure 3: Therapeutic potential of pumpkin^2–7^ (Antidiabetic: reprinted from ref 2 Copyright 2020 with permission from Elsevier; Treat Malnutrition: adapted with permission from ref 3 published by Springer 2017; Treat Liver diseases: adapted with permission from ref 4 published by Springer 2014; Anticancer: adapted from ref 5 published by MDPI 2021 under an open access Creative Common CC BY license; Prevent from brain damage: adapted from ref 6 published by Rutgers 2018; Anti-Inflammatory: adapted from ref 7 published by IntechOpen 2022 under an open access Creative Common CC BY license).
